# Rat Heterotopic Heart Transplantation Model to Investigate Unloading-Induced Myocardial Remodeling

**DOI:** 10.3389/fcvm.2016.00034

**Published:** 2016-10-19

**Authors:** Xuebin Fu, Adrian Segiser, Thierry P. Carrel, Hendrik T. Tevaearai Stahel, Henriette Most

**Affiliations:** ^1^Department of Cardiac and Vascular Surgery, Inselspital University Hospital, Berne, Switzerland

**Keywords:** heterotopic heart transplantation, myocardial unloading, reverse remodeling, heart failure animal model, myocardial recovery

## Abstract

Unloading of the failing left ventricle in order to achieve myocardial reverse remodeling and improvement of contractile function has been developed as a strategy with the increasing frequency of implantation of left ventricular assist devices in clinical practice. But, reverse remodeling remains an elusive target, with high variability and exact mechanisms still largely unclear. The small animal model of heterotopic heart transplantation (hHTX) in rodents has been widely implemented to study the effects of complete and partial unloading on cardiac failing and non-failing tissue to better understand the structural and molecular changes that underlie myocardial recovery. We herein review the current knowledge on the effects of volume unloading the left ventricle *via* different methods of hHTX in rats, differentiating between changes that contribute to functional recovery and adverse effects observed in unloaded myocardium. We focus on methodological aspects of heterotopic transplantation, which increase the correlation between the animal model and the setting of the failing unloaded human heart. Last, but not least, we describe the late use of sophisticated techniques to acquire data, such as small animal MRI and catheterization, as well as ways to assess unloaded hearts under “reloaded” conditions. While giving regard to certain limitations, heterotopic rat heart transplantation certainly represents the crucial model to mimic unloading-induced changes in the heart and as such the intricacies and challenges deserve highest consideration. Careful translational research will further improve our knowledge of the reverse remodeling process and how to potentiate its effect in order to achieve recovery of contractile function in more patients.

## Introduction

Heart failure (HF) is a life-threatening disorder worldwide. In end-stage HF patients, there are no curative treatment alternatives to heart transplantation that substantially improve survival. For instance, left ventricular assist device (LVAD) implantation can provide powerful circulatory support and improve the survival and quality of life compared with the existing pharmacological treatment regimen (1). At the same time, these devices result in volume unloading of the ventricle, which results in reversal of pathological processes, termed reverse remodeling, to an extent that can allow functional recovery of the failing myocardium. In a subset of patients, this allowed explantation of the device and prolonged freedom from HF (2, 3). But, prolonged periods of LVAD-induced unloading are also deemed detrimental because they lead to cardiac atrophy and increased stiffness of the myocardium (4). The complex response to mechanical unloading necessitates a reproducible small animal model of mechanical unloading for use in the laboratory.

As such, the rat heterotopic heart transplantation (hHTX) model has been set up and optimized to study mechanical unloading (5–7). This review focuses on adaptations of the hHTX model to more closely resemble the human setting, and it summarizes the recent advances of preclinical research to understand unloading-induced changes in normal and failing myocardium and finally specifies optimization of assessment modalities.

## Heterotopic Heart Transplantation Models and Their Limitations

**Figure 1:**
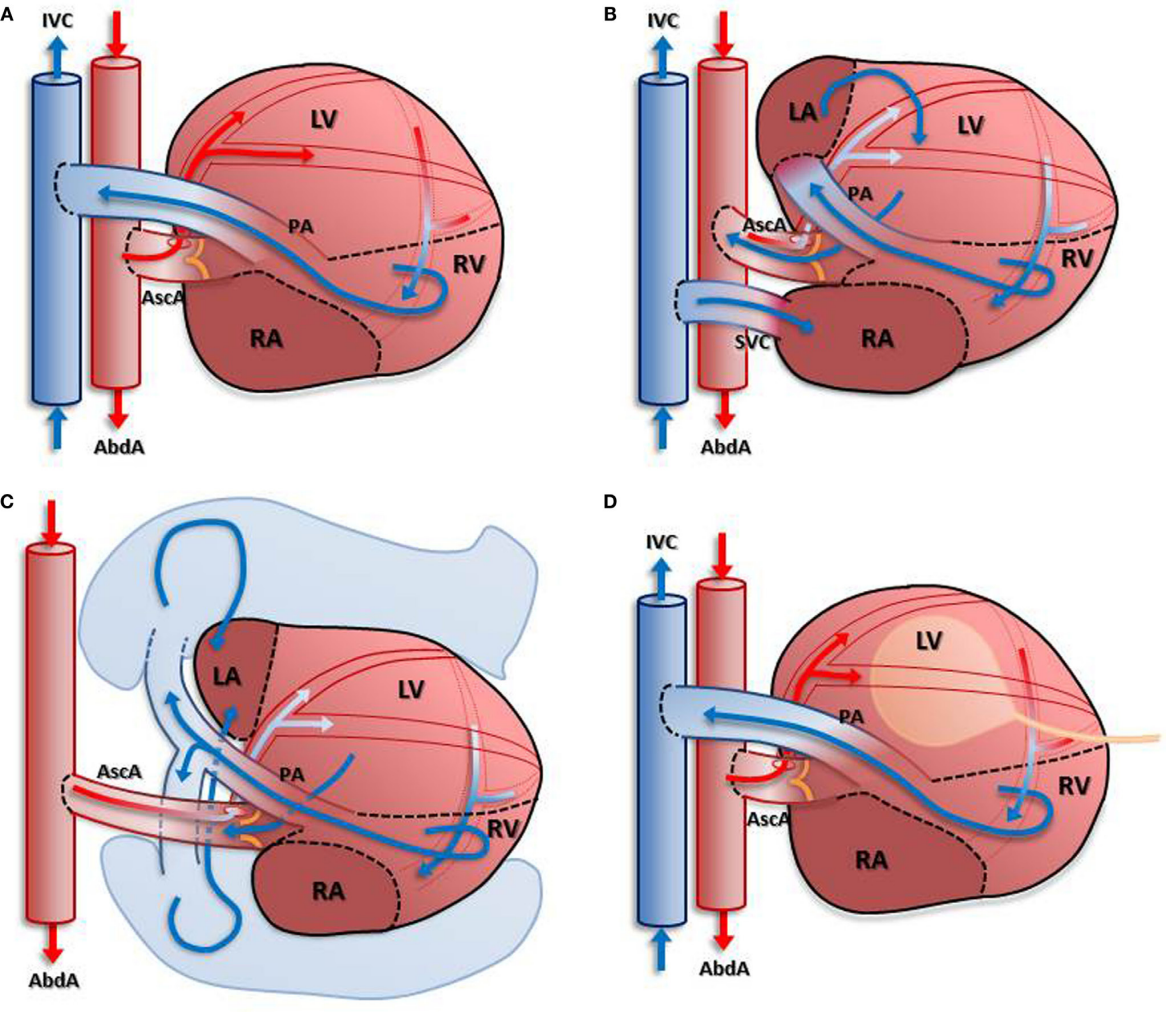
**Depiction of different models of unloading compared with completely unloaded left ventricle**. In **(A)**: blood enters the coronary circulation antegradely *via* the anastomosis created between the donor’s ascending aorta and the recipient’s abdominal aorta. Subsequently, venous blood from the coronary sinus is ejected into the recipient’s inferior vena cava *via* the pulmonary artery anastomosis. Due to the closed aortic valve, the left ventricle is unloaded in this model. Partial load is achieved in models depicted in **(B–D)** either by return of the venous coronary blood flow into the left atrium **(B)** with an additional anastomosis or using the pulmonary circulation **(C)**. A different approach is depicted in **(D)**: insertion of a latex balloon *via* the apex allows differential loading of the left ventricular myocardium.

Whether the degree of mechanical unloading influences the beneficial effects to the heart is under debate: therefore, the idea of limiting the duration and intensity of ventricular unloading to prevent detrimental effects counteracting efficient reverse remodeling has been proposed. Various adaptations of the hHTX small animal model to make partial volume unloading possible have been developed and applied (Figure 1), allowing comparison with complete unloading.

### Partial vs. Complete Unloading

#### Completely Unloaded Left Ventricle

The completely unloaded left ventricle is achieved by using the “standard” cardiac heterotopic transplantation technique: an isogenic “donor” heart is transplanted infrarenally by anastomosing the ascending aorta of the donor end-to-side to the abdominal aorta of the “recipient,” within an ischemic time of ∼20 min. The pulmonary artery of the transplant is anastomosed end-to-side to the inferior vena cava. Coronary artery flow antegradely perfuses the myocardium, the venous blood returns *via* the right ventricle into the recipients’ circulation, whereas the left-sided cavities remain unloaded in this spontaneously beating graft. Thus, the left ventricle of heterotopically transplanted hearts is, in essence, completely unloaded (Figure 1A). Although this model has been applied for decades to study mechanical unloading, a recent report indicated that there is a high variability of left ventricular volume-loading status, compromising the results. The authors report observations from some “unloaded” hearts exhibiting considerable ventricular filling and ejection (8).

#### Partially (Un)Loaded Left Ventricle

A partially unloaded heart model can be achieved by various methods. Here, we describe the most frequently and successfully used ones, also illustrated in Figure 1:

(a) Partial volume-loading model: the aorta of the donor is anastomosed to the abdominal aorta of the recipient, the pulmonary artery of the donor to the left atrium of donor, and superior vena cava of donor to inferior vena cava of recipient (9). This model (Figure 1B) returns some of the coronary circulation blood flow to the left ventricle *via* the additional anastomosis, thus allowing for a partial loading of the LV. The major disadvantages of this model are requirement of an additional anastomosis, resulting in longer ischemic time, and the mixture of desoxygenated blood in the arterial coronary circulation (10), which has been shown to cause LV dysfunction (10).
(b) Heterotopic heart–lung transplantation: the ascending aorta “donor” heart is anastomosed to the “recipients” abdominal aorta, the “donor” heart’s venae cavae are ligated, and the pulmonary artery and pulmonary veins are intact (7). In this model (Figure 1C), the left ventricle ejects blood returning from the pulmonary circulation and the aortic valve opens intermittently. Although there is no available data on the oxygen saturation in the blood entering the coronary circulation to date, it can be assumed that this technique results in a similar mix of oxygenated and desoxygenated blood as in the other known volume-loaded models.
(c) Balloon technique: in a classical heterotopically transplanted heart, the left ventricular cavity is filled with a small balloon of certain volume to create isovolumic load (11). This model (Figure 1D) requires creation of an arterio-venous fistula to increase caval oxygen saturation and may result in venous thrombosis and thromboembolism.

A load-dependent relationship exists regarding the left ventricular diameters (posterior wall thickness, diastolic interventricular septum, and left ventricular end-diastolic dimension) (7). This also affects LV fractional shortening and ejection fraction and was further underscored in measurements of the pressure–volume relation. Significantly, more myocardial atrophy and fibrosis were observed in completely unloaded hearts, whereas concentrations of cytokines and matrix metalloproteinases were comparable in both unloading conditions.

Similarly, chronic partial unloading but not complete unloading restored beta-adrenergic responsiveness and reversed receptor downregulation in failing rat hearts in a different study (12).

The degree and duration of mechanical load variation caused by hHTX also regulates cardiomyocyte Ca^2+^ handling (13): Ca^2+^ release synchronicity was reduced at 8 weeks in partially unloaded hearts only. Moderate partial unloading had milder effects compared with complete unloading in cell volume reduction and did not cause depression and delay of the Ca^2+^ transient, increased Ca^2+^ spark frequency, or impaired t-tubule and cell surface structure.

### Unwanted Effects and Limitations of the HTX Model

Heterotopic transplantation of a heart elicits a number of effects that can influence the obtained results and have to be taken into account when evaluating the unloaded heart.

#### Systemic Effects

In clinical application, implantation of an LVAD improves whole body perfusion and restores neurohormonal activation, this being one of the main reasons for the benefit to patients (14). These systemic beneficial effects cannot be mirrored or observed in experimental heterotrophic transplantation models. It could be argued that the transplantation of a failing heart into a healthy recipient with normal levels of circulating neurohormones and intact function of all organs can serve as an example for the normalization occurring in the HF patient on circulatory support systems.

#### Denervation and Heart Rate

Denervation causes beneficial cardiac changes and the beneficial effects, such as attenuation of remodeling and functional improvement warrant investigation if this approach can potentially be applied to treat HF (15). In the HTX model, the effect of denervation on the transplanted heart cannot be excluded or differentially assessed, as one difference from the clinical setting, in which the unloaded heart remains innervated. Therefore, it is critical to take the reduced and somewhat fixed heart rate of the grafts into account when assessing the functional capacity of the heterotopically transplanted heart.

#### Ischemia–Reperfusion Damage

The exposure to I/R is inevitable during HTX in all models, reported ischemic times are around 30 min for the conventional hHTX (10). During hHTX, ischemic time of each heart is different. However, there were no special concerns about the ischemia/reperfusion injury reported in most published papers. To overcome technical difficulties, which could prolong graft ischemia, several improvements have been proposed, such as a cuff design system, which allows easy manipulation to manage the transplantation with an ischemic time of <11 min on average (16).

#### Immune Reaction/Rejection of the Allograft

After its first description in 1958 (5), the HTX model was commonly used for studies assessing transplantation immunology (10). Although all studies that examine the effect of left ventricular unloading use in-bred, isogenic rat strains, for instance Lewis rats, the biological effects of this transplantation have not been extensively studied.

#### Lack of Passive Resistance

The introduction of the heart into an abdominal environment, which is quite different from original location in the chest, causes concern, although the consequences remain unknown to date. The passive resistance of pericardial fluid and a pericardial sack surrounding the heart is known to cause ventricular interdependence, which must be disrupted in the graft with unknown hemodynamic consequences (17).

## Unloading-Induced Effects: Good and Bad

### Mechanical Unloading Leads to Adaptation of Cardiac Mass and Cardiomyocyte Size

**Figure 2:**
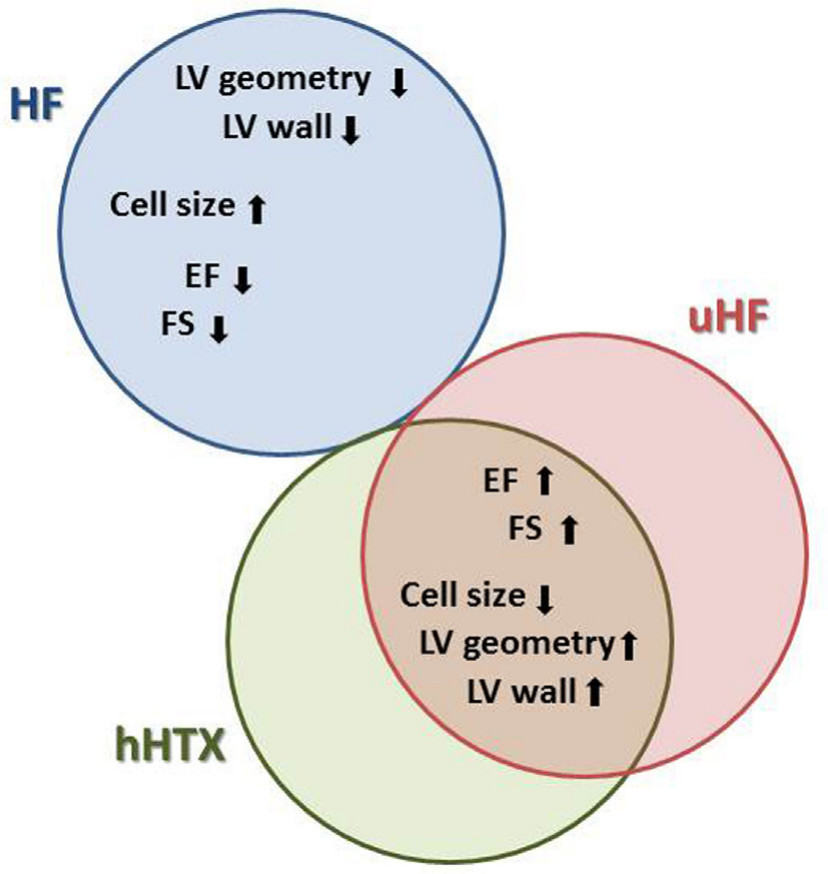
**This diagram illustrates parallel changes in heart failure (HF), unloaded normal rat hearts (hHTX), and unloaded failing myocardium either from human hearts supported with assist devices or from animal experiments (uHF), as well as differences between these stages**. With respect to cardiac structure and myocardial cell size, for instance, changes elicited by unloading are similar in normal and failing hearts, a reversal of those seen in failing myocardium.

In clinical practice, as one of the many features of LVAD supported failing hearts, a shrinkage of the enlarged heart is consistently observed (18). This reduction of the overall cardiac mass is the most distinct and consistent phenomenon (18), and it has been replicated and investigated in the hHTX model (Figure 2).

In hypertrophic and failing hearts, unloading reverses pathological enlargement of cardiac myocytes and normalizes myocardial mass, while in heterotopically transplanted normal hearts, we find reduction of cellular diameters as well as a relative decrease of myocytes compared with connective tissue, this being termed atrophy (19, 20). While the normalization of hypertrophy in the failing unloaded heart can be explained by the disappearance of increased wall stress, which goes along with the initiation of a similar gene expression program in both hypertrophy and after unloading (21), the atrophy following unloading of a normal rat heart must be an active adaptation to the loss of volume load, which includes autophagy and apoptosis (22).

### Apoptosis in Mechanically Unloaded Hearts

**Figure 3:**
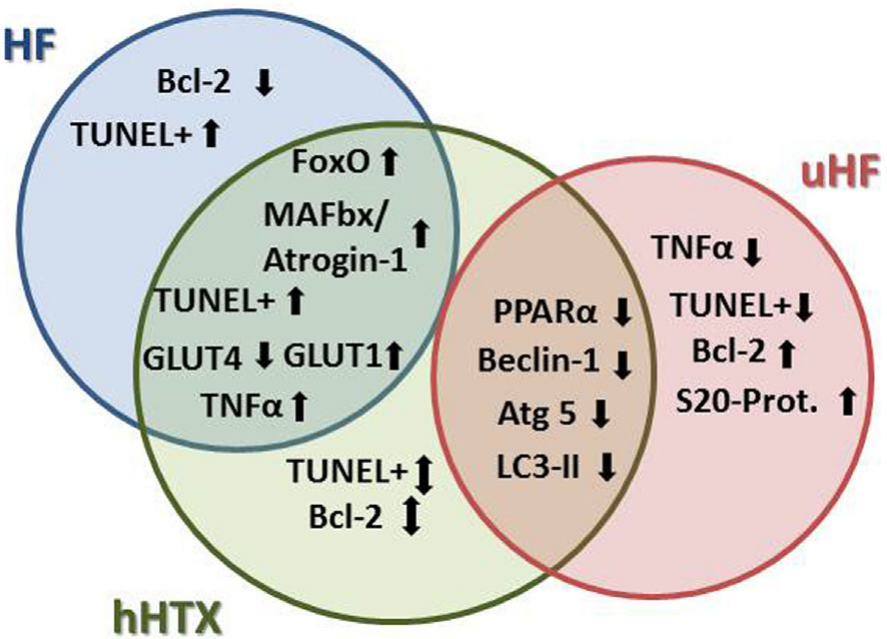
**Regarding atrophy, apoptosis, and autophagy, we find more similarities between unloaded normal rat hearts and failing hearts, since apoptosis and atrophy are induced in HF, but were also seen in rat myocardium after hHTX**. On the other hand, markers of autophagy were induced in a similar way in hHTX and unloaded failing hearts, indicating regulation of changes that are possibly counteracting each other in hHTX.

A progressive decline in LV function has been linked to loss of nearly one-third of all cardiomyocytes as a result of apoptosis in cardiomyopathy, both in human (23) and in animal HF, with both proapoptotic and antiapoptotic pathways being activated in advanced HF (24). Basic and translational research shows contradictory findings on the effects of LVAD unloading on apoptosis to date (25), as programmed cell death constitutes part of the underlying disease with heterogenic pathology (Figure 3).

In a study investigating mechanically unloaded dilated cardiomyopathy (DCM) hearts in the hHTX model, the TUNEL-positive myocyte count indicated that there was no difference after 2 weeks of unloading. However, 4 weeks after unloading, the number of TUNEL-positive myocyte in DCM-unloaded group was higher than that in the DCM group (26). The underlying mechanisms are not clear. It was reported that there is a significant increase in caspase-3 mRNA expression and activity after mechanical unloading in a normal unloaded heart (22, 27, 28). In addition, relatively late expression changes in genes involved in the apoptosis process were found at the protein level. Increased protein expression of bax resulted in a higher apoptotic index (bax/bcl-2 ratio) after 30 and 60 days of unloading. Expression of the antiapoptotic protein bcl-2 was only decreased at the mRNA level, but not at the protein level (22).

In the small animal model, the rate of apoptotic cell death is reduced in unloaded failing hearts (29), as a result of diminished toxic catecholamine overstimulation and improved energy metabolism. But, somewhat astonishingly, it was found that unloaded normal hearts exhibit increased apoptosis after prolonged unloading time (22). This speaks toward a late induction of apoptosis as the result of disuse and might indicate that the beneficial changes that lead to reverse remodeling in the failing heart could be counterbalanced by detrimental reduction of cardiomyocytes with prolonged periods of unloading.

### Autophagy

Autophagy was induced during regression of cardiac hypertrophy, as evidenced by an increase in microtubule-associated protein 1 light chain 3 (LC3)-II protein level. Cardiac-specific Atg5-deficient (CKO) mice showed significantly less regression of cardiac hypertrophy compared with normal controls (30). Sadoshima’s group found that the FoxO family of transcription factors is involved in autophagy during mechanical unloading of the heart. Transgenic mice with cardiac-specific overexpression of FoxO1 exhibited smaller hearts and upregulation of autophagy (31). Autophagy-mediated degradation also seemed necessary for regression of cardiac hypertrophy during ventricular unloading. By hHTX of normal rat hearts, Cao et al. found autophagy was activated *via* transcription factor FoxO3 and bnip3 in unloaded hearts (32), which went along with the reduction of mitochondrial proteins.

Razeghi et al. indicated that both protein synthesis and degradation were activated in heterotopically transplanted rat hearts (19). Atrophic changes seemed to change the balance of protein synthesis and degradation and inhibition of the involved signaling pathway downstream of mTOR by Rapamycin led to augmented atrophy even (19). Unloading seems to initiate processes leading to a new steady state after 30 days (19, 20), at which a maintenance of cardiac mass seems to be reached.

Ligases of the ubiquitin system, such as MuRF1 and Atrogin-1/MAFbx, are also involved in the atrophic changes (33) *via* autophagy. Protein degradation was not significantly altered in unloaded hearts lacking Atrogin-1/MAFbx (Atrogin1/MAFbx^−/−^), but protein synthesis was dramatically elevated in atrogin1/MAFbx-deficient cardiomyocytes. Calcineurin and NFAT activity were significantly increased in Atrogin-1/MAFbx^−/−^ CMs (33).

In human failing hearts, mechanical unloading resulted in a decrease of markers of autophagy, suggesting that autophagy may be a maladaptive mechanism in the failing hearts, which is attenuated by LVAD support (34). This is also supported by the knowledge that autophagy plays an essential role in mediating regression of hypertrophy during unloading of the heart (31, 35). Chronic activation of autophagy can lead to excessive degradation of critical cellular proteins and organelles, which will induce cell death. The complex role of autophagy in the unloaded failing heart needs to be further investigated.

The term atrophy currently denominates a “side-effect” of mechanical unloading. Although primarily on the basis of animal data, the notion that chronic LVAD-induced unloading will result in atrophy has dominated the clinical HF field, and antiatrophic drugs have been used to enhance the cardiac recovery potential in LVAD patients (36).

### Antiatrophic Pharmacological Therapy with Mechanical Unloading

Since the treatment with LVAD alone is not sufficient in eliciting and exploiting the potential for recovery in most chronic HF patients, adjunct complimentary and combination therapy have been proposed (37). Clenbuterol has been investigated in the small animal hHTX model as well as in clinical trials: a rationale for a potential beneficial antiatrophic effect was the known anabolic capacity of Clenbuterol and other β2-adrenergic agonists. In the hHTX model, it significantly increased the weight of the recipient rat hearts, but did not prevent unloading-induced LV atrophy in the transplanted heart (38, 39). There was no difference in LV developed pressure between the Clenbuterol-treated and control group. However, Clenbuterol treatment improved gene expression (SERCA2a, beta-MHC) and beta-adrenergic responsiveness and potentially prevented myocardial apoptosis (38). Thus, the combination of Clenbuterol with mechanical unloading seemed promising in HF. In the small animal model, adjunct treatment with Clenbuterol increased sarcomere shortening by normalizing the depressed myofilament sensitivity to Ca^2+^ in cardiomyocytes. Clenbuterol limited unloading-induced reduction of cell size, which further supported the rationale for its use to limit unloading-induced myocardial atrophy (39). Furthermore, Clenbuterol, if combined with a β1 blocker (Metoprolol) and mechanical unloading enhanced functional recovery *via* improvement in ejection fraction (EF) while preventing Clenbuterol-induced hypertrophy and tachycardia. But, at closer observation, the results of this investigation were not consistent: during mechanical unloading, the combined pharmacological therapy was less beneficial than either monotherapy in preventing myocardial atrophy. Metoprolol, not Clenbuterol, prevented unloading-induced myocardial atrophy, with increased atrophy occurring during combined therapy. Unloading-induced recovery of Ca^2+^ transient amplitude, speed of Ca^2+^ release and sarcoplasmic reticulum Ca^2+^ content was enhanced equally by each monotherapy, but these benefits, together with Clenbuterols enhancement of sarcomeric contraction speed, and unloading-induced recovery of Ca^2+^ spark frequency disappeared during combined therapy (40).

Promising data from several clinical trials of the London Harefield group around Magdi Yacoub have not lived up to its promise, largely, because the results could not be replicated to the full extent at other centers (3). A potential explanation would be that atrophy is not necessarily associated with either recovery/regression of the failing heart (41) or deleterious effect of unloading (22). If so, atrophy cannot be used as an indicator for recovery or maladaptation. Further detailed mechanisms need to be investigated. Of course, atrophy of the unloaded normal heart cannot simply explain the dynamics in LVAD supported failing hearts, since it cannot mimic the confounding effects of specific pathologies leading to HF.

Early preclinical data exist for beneficial effects of innovative pharmacological and cell therapy in the setting of unloading-induced myocardial recovery: ivabradine, combined with mechanical unloading was shown to reverse myocardial fibrosis and enhance the restoration of deranged E–C coupling (42) in one study using the small animal hHTX model. Also, hHTX was combined with cell therapy, where mechanical unloading in mice *via* hHTX was able to increase survival rate and differentiation of cardiac stem cells after implantation (43). Another agent previously successfully used in HF therapy, thyroid hormone (T3) at physiological doses restored depressed contractile reserve and impaired calcium handling of cardiac myocytes from chronically unloaded hearts (44, 45). These findings require more rigorous and in-depth testing, but reveal the worth of the hHTX model to provide a great opportunity to investigate several potential treatments with mechanical unloading. This model strongly impacts preclinical studies to validate ideas and illustrate potential effects with new combinations.

### Metabolic Changes in Unloaded Hearts

In theory, when the heart is unloaded, metabolic alterations reflect the decreased energy demand of the cardiac muscle and also its compromised capacity to generate sufficient amounts of adenosine triphosphate (ATP). Similarly, the failing heart develops alterations of metabolism, such as compromised glucose uptake and fatty acid oxidation as one of the maladaptive features distinctive of HF. If the mechanical unloading of myocardium can ameliorate or aggravate metabolic changes in the failing heart, is a key question to be addressed.

In failing human hearts, metabolic effects lead to impaired substrate utilization and subsequent disturbance of energy homeostasis (46). Comparison of myocardial samples taken before and after LVAD implantation revealed that unloading improved decreases in key transcription factors and their target enzymes critical to cardiac metabolism (47). Whether the unloaded heart can keep the flexibility to respond to different energy demand needs to be further investigated.

### Generation of Energy Substrates

Glucose and fatty acids are both main energy substrates for the heart. In the healthy adult myocardium, β-oxidation of fatty acids accounts for about 70% of the ATP generation (48). However, per molecule of ATP, oxidation of glucose requires less oxygen, thus increasing efficiency up to 25%. In hypertrophied and failing hearts, a characteristic switch of Myosin heavy chain isoforms occurs and limits performance. This switch in favor of α-MHC, called fetal expression pattern, was also found in mechanically unloaded hearts (21). Therefore, it was proclaimed that a correlation exists between the adaptation of energy-consuming and energy-producing pathways in both hypertrophy and unloading (49).

Further supporting this notion, it was reported that mechanical unloading led to remarkably diminished glucose uptake (7, 9) in completely unloaded myocardium, but not in partially unloaded LV myocardium (9), although these data were not reproduced yet. One study claimed that glucose transporter type 1 (GLUT-1, fetal type) mRNA increased in the unloaded heart with no changes in glucose transporter type 4 (GLUT-4, adult type) mRNA (Figure 3). The other report discovered that GLUT-4 was decreased in unloaded hearts; however, GLUT-1 remained the same as the control (9, 21). GLUT-4 is the main isoform found in the normal adult heart. The decrease of GLUT-4 in the unloaded hearts reflects the decrease in energy needs.

Although it looks like alterations in transcription patterns of metabolic genes account for the substrate preference shift from fatty acids to glucose in the atrophic heart, the mechanism of this shifting is not completely understood. For instance, there is no convincing evidence for increasing expression of glycolytic enzymes in unloaded cardiomyocytes to support previous hypotheses. Whether glycolysis is enhanced or not in unloaded heart is currently not known.

### Mitochondrial Detrimental Effects

The precise temporal activation of metabolic effects during mechanical unloading is largely not clear to date. It was shown that mitochondrial protein expression is diminished in unloaded hearts with ongoing atrophy (50). During atrophic myocardial changes, mitochondrial damage caused by increased mitochondrial uncoupling protein activity or increased production of reactive oxygen species reduces ATP production and leads to metabolic dysfunction. Consistent with this, mechanical unloading of the failing heart does not restore the transcript levels of metabolic genes with the notable exception of uncoupling protein 3 (UPC3) (51). On the other side, UPC2 and mitochondrial transmembrane proteins that create a proton leak which uncouples ATP synthesis from the electron transport chain is decreased in pathological remodeling (52). In addition, during cardiac atrophy, expression of peroxisome proliferator-activated receptor-α (PPAR-α) and PPAR-coactivator-1α (PCG-1α) activity are decreased (53). This reduction predicates reduced mitochondrial gene expression and reduced oxidative capacity. And this could be another reason for the metabolic crisis in atrophied hearts.

AMP-activated protein kinase (AMPK), which is the metabolic master switch regulating several intracellular systems including the cellular uptake of glucose, the β-oxidation of fatty acids, was recently investigated in heterotopically transplanted hearts. A reduced ATP-to-AMP ratio will activate AMPK signaling in cardiomyocytes. In a study investigating a role for AMPK in cardiac autophagy and protein degradation induced *in vitro* by starvation, it was shown that AMPK-induced expression of ubiquitin ligases MuRF1 and Atrogin-1, which resulted in cardiomyocyte dysfunction (49). While these ligases are also strongly involved in unloading-induced shrinking of the heart, the role of AMPK remains unclarified to date.

## Ca^2+^ Cycling and Contractile Function in the Unloaded Cardiomyocyte

### Enhanced Ca^2+^ Influx through Cardiac L-Type Ca^2+^ Channels

Excitation–contraction coupling in cardiomyocytes requires appropriate Ca^2+^ handling and sarcomeric protein expression and assembly. Intracellular Ca^2+^ transients are pivotal to electromechanical coupling and to cardiac plasticity (54–56). Whether Ca^2+^ transients are changed will critically influence contractile function in mechanically unloaded hearts.

Although certain decreased expression of proteins implicated in Ca^2+^ regulation, the shape of the systolic Ca^2+^ transient remains unaltered after atrophic transformation (57, 58). Data on the particulars of Ca^2+^ transients, i.e., the amplitude, the diastolic Ca^2+^ concentration, as well as the time course of the Ca^2+^ transients, are not consistent: some found them not to be significantly different between unloaded hearts and controls (58), while others found that the peak amplitude of the whole-cell Ca^2+^ transient was significantly smaller after hHTX unloading (59). The variance of time-to-peak of the Ca^2+^ transients was significantly increased in unloaded cardiomyocytes, suggesting that the synchronicity of Ca^2+^ release was disrupted. L-type Ca^2+^ current density was unaffected, and inactivation was delayed (59).

In myocytes isolated from atrophic hearts, the amplitude of transient inward current (*I*_ti_) (peak *I*_ti_) was significantly lower than in control myocytes (10% in endocardial and 50% in epicardial myocytes). Sarcoplasmic reticulum (SR) Ca^2+^ content was reduced by ~55% in unloaded hearts (58). Atrophic cardiac myocytes showed a 45% lower frequency of spontaneous diastolic Ca^2+^ sparks. Quantification of spontaneous Ca^2+^ release in control and thapsigargin-treated cardiac myocytes revealed that the partial depletion of the SR caused a ~65% lower mean Ca^2+^ spark frequency and a 2.5-fold higher fraction of cells with a very low spark activity. Ryanodine receptor expression was significantly increased (130% of control) in the atrophic myocardium, whereas its phosphorylation at Ser_2814_ was unaltered (58). These data suggest that, in early cardiac atrophy induced by mechanical unloading, an augmented sarcolemmal Ca^2+^ influx through LTCC compensates for a reduced systolic SR Ca^2+^ release to preserve the Ca^2+^ transient amplitude. This interplay involves an electrophysiological remodeling as well as changes in the expression of cardiac ion channels. In this thorough study, it was also hypothesized that the unaffected Ca^2+^ transients in spite of a decreased SR Ca^2+^ content could be a consequence of an unaltered absolute amount of Ca^2+^ released from the SR during systole. The Ca^2+^ transient in atrophic cardiac myocytes must be maintained by a compensatory mechanism other than an increased fractional release from the SR.

Another explanation for unchanged Ca^2+^ transients in the presence of a reduced SR Ca^2+^ load is an increased Ca^2+^ influx during the action potential (AP). Schwoerer et al. indicate that in atrophic cardiac myocytes Ca^2+^ influx through the L-type Ca^2+^ channel (LTCC) is augmented both by an increased electrical driving force caused by prolonged APs and by an increased membrane abundance of Ca^2+^ channel subunits.

### The Action Potential in Unloaded Cardiomyocytes

Unloaded rat hearts displayed markedly prolonged APs with a pronounced plateau phase (58). Known mechanisms underlying prolonged APs are often changes in the repolarizing K^+^ current, in particular, of the transient outward K^+^ current (*I*_to_). The reduction of *I*_to_ can be associated with significantly lower levels of Kv4.2 and Kv4.3 mRNAs in epicardial myocytes and of KChIP2 mRNA in endocardial myocytes. Current clamp recordings revealed prolonged APs in endocardial as well as epicardial myocytes, which were associated with a twofold to fourfold higher sarcolemmal Ca^2+^ influx during the AP. In addition, Caveolin 1.2 subunits, which form the pore of the LTCC, were upregulated in atrophic myocardium.

### Mechanical Unloading Reverses Certain Electrophysiological Features of HF

**Figure 4:**
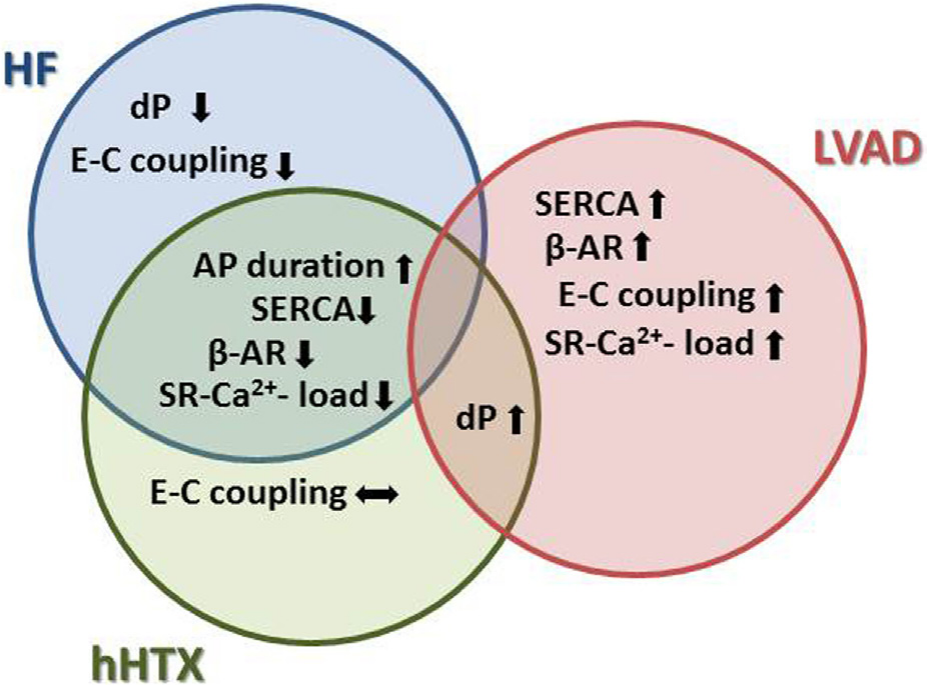
**Effects of unloading to sarcomeric function and Ca^2+^ cycling indicate more detrimental changes in unloaded normal rat hearts, similar to remodeling in HF**. For instance, Ca^2+^ cycling and adrenergic signaling are negatively impacted in both HF and after hHTX, as opposed to the reverse remodeling process seen after LVAD implantation.

Ca^2+^-induced Ca^2+^ release (CICR) is critical for effective contraction in cardiomyocytes. The transverse (t)-tubule system guarantees the proximity of the triggers for Ca^2+^ release (LTCC, dihydropyridine receptors) and the SR Ca^2+^ release channels (ryanodine receptors). Transverse-tubule disruption occurs early in HF, and the typically reduced t-tubule density was reversed by unloading (59): both the decreased density and also the deterioration in the regularity of the t-tubule system were reversed by mechanical unloading (Figure 4).

In a follow-up study, Ibrahim and colleagues evaluated L-type Ca^2+^ current density in unloaded failing hearts, measured using whole-cell patch clamping, and observed that it was reduced in HF, but this was unaffected by unloading in contrast to the normal unloaded hearts (60). Scanning ion conductance microscopy revealed reappearance of normal surface striation in unloaded failing hearts. Electron microscopy showed recovery of normal t-tubule microarchitecture in that same study. The variance of the time-to-peak of the Ca^2+^ transient, an index of CICR dyssynchrony, was increased in HF and normalized by unloading. The increased Ca^2+^ spark frequency observed in HF was reduced *via* unloading. These results were explained by the recoupling of orphaned RyRs in HF, as indicated by immunofluorescence. Taken together, the authors concluded that mechanical unloading of the failing heart reversed the pathological remodeling of the t-tubule system and improved CICR (60).

Following a different therapeutic adjunct to unloading, it was suggested that physiological treatment dose of thyroid hormone rescued the impaired myocyte relaxation and depressed contractile reserve at least partially through the restoration of phospholamban (PLB) protein levels and its phosphorylation state in chronically unloaded hearts (44). In vehicle-treated animals, myocyte relaxation and Ca^2+^ decay were slower in unloaded hearts than in recipient hearts. Myocyte shortening in response to high Ca^2+^ was also depressed with impaired augmentation of peak-systolic Ca^2+^ influx in unloaded hearts compared with control. In vehicle-treated rats, protein levels of PLB were increased by 136% and the phosphorylation of PLB decreased by 32% in unloaded hearts. In the thyroid hormone-treated animals, these features leading to depressed contractile reserve in myocytes from unloaded hearts were all returned to normal levels (44).

Takaseya and colleagues reported that mechanical unloading increases SR Ca^2+^ ATPase and improves Ca^2+^ handling and contractility in rats with doxorubicin-induced cardiomyopathy. These beneficial effects of mechanical unloading were not observed in normal unloaded hearts (61).

Summarizing these data, one might conclude that our current knowledge indicates a beneficial role for unloading on Ca^2+^ cycling and E–C coupling in failing myocardium, while the atrophic changes induced in normal hearts *via* hHTX lead to perturbations deleterious to its Ca^2+^ homeostasis.

### Unloading-Induced Recovery of Adrenergic Responsiveness

The failing myocardium is characterized by decreased force generation and slowed relaxation as a consequence of depressed response to β-adrenergic stimulation. Studies comparing failing and unloaded failing human hearts confirmed a certain improvement of β-adrenergic response and subsequent contractile properties many times over (62, 63). The underlying reversion of pathological adrenergic signaling and responsiveness of the contractile apparatus of cardiomyocytes can be studied in the small animal model (6), but a detailed and accurate assessment of density and activity of β-adrenergic receptors and coupling to adenylyl cyclase is still missing.

With particular interest given to unloading time, Oriyanhan and colleagues studied functional recovery in a rat ischemic HF model with unloading times of 2, 4, and 8 weeks and found that the maximal inotropic response occurred at 4 weeks in isolated papillary muscles from unloaded failing hearts (29). In parallel to the approach in humans, Soppa and colleagues reported therapeutic potential on Clenbuterol on unloaded failing rat hearts after 7 days: the sensitivity to Ca^2+^ and sarcomere shortening were superior in Clenbuterol-treated hearts, but the density and activity of β-adrenergic receptors was not reported (39).

In a study using a partially unloaded hHTX model, developed tension of posterior papillary muscle was significantly increased in the partially unloaded group compared with the HF group (64). The mRNA expression of brain natriuretic peptide (BNP), SERCA2a, and β1- and β2-adrenergic receptors (β1- and β2-AR) in LV tissue was almost normalized in partially unloaded hearts.

## Extracellular Matrix Reorganization with Mechanical Unloading

The extracellular matrix (ECM) of cardiac tissue is comprised mainly of collagen type I, elastin, proteoglycans, and glycoproteins and is integral to the maintenance of tensile strength and myofibrillar organization of cardiac muscle (65). Because intimate connections between the cardiomyocytes and ECM are required to coordinate contraction of the myocardium, cardiac structural rearrangement cannot be limited to the cardiomyocytes; decreased cardiomyocyte volume alters the relationship between cells and the ECM. Changes in the ECM composition and collagen turnover, largely a result of balancing matrix metalloproteinases (MMP-1, -2, and -9) and tissue-inhibitor of matrix metalloproteinases (TIMP-2 and -3) activation, after mechanical unloading of failing and normal hearts have been characterized.

**Figure 5:**
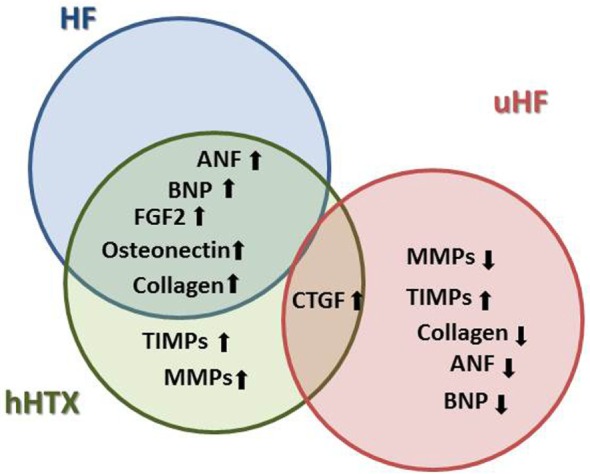
**Changes in the extracellular matrix plays a crucial role in the development and progression of HF, and key elements of pathological remodeling such as increased neurohormone and growth factor expression are also found in the small animal model of hHTX, whereas these changes were reversed in LVAD-unloaded human failing myocardium**.

Observations in failing human hearts indicate a beneficial regression of fibrosis and myocardial stiffness in hearts unloaded *via* LVAD (66, 67), as one of the reasons for partial recovery of function (Figure 5). A correlation between changes in HF patient myocardium after LVAD, such as increases in connective tissue growth factor-, alpha 3 collagen-, and osteonectin-mRNA, with results found in the unloaded normal rat hearts may be established (22, 67).

After unloading of rat hearts with ischemic HF *via* coronary artery ligation, mRNA levels of MMP-1, -2, and -9 were normalized, and TIMP-2 and TIMP-3 were upregulated compared with HF (68). This led to a normalization of the MMP/TIMP ratio, indicative of beneficial reverse remodeling. Quite contrary, in normal unloaded hearts, mRNA expression of MMP-2 and MMP-9 increased on the 7th and 14th days after heart transplantation and peaked on the 14th day compared with the control group. The gelatinase activity as well as myocardial ECM deposition significantly increased with duration of unloading. Although both mRNA and protein levels of TIMP-1 and TIMP-2 were also increased, it was proclaimed that mechanical unloading may lead to adverse remodeling of the ECM of the left ventricle, the underlying mechanism being an imbalance of the MMP/TIMP system, especially taking into account the remarkable upregulation of TIMPs in the pressure and volume unloaded heart (69). Additionally, we recently found that mRNA expression level of fibroblast growth factor (FGF-2) was significantly increased after prolonged unloading (22), potentially further promoting fibrosis of the myocardium. Furthermore, in the small animal model of hHTX, after left anterior descending artery ligation induced HF, Oriyanhan et al. found that myocardial fibrosis of the failing heart increased further after unloading (29). Similar results were repeatedly found with myocardial infarction or DCM-induced HF and unloading hHTX models (26, 64).

On the other hand, expression of the fetal genes, atrial natriuretic factor (ANF) or brain natriuretic peptide (BNP), which are upregulated not only in HF but also in atrophic myocardium, has been shown to oppose myofibroblast activity. Activated myofibroblasts enhance the synthesis and secretion of type I fibrillary collagen (65), an increased expression of matrix proteins after unloading was observed to cause myofibrillar disarray and excessive ECM, which negatively affects cardiac function causing tissue stiffness (70). These findings are supporting the notion that the initial response to hemodynamic unloading may be cardioprotective in nature and that a beneficial effect of unloading may be time dependent (71).

However, detailed ECM configuration affecting cardiomyocytes, cardiac fibroblasts, and cardiac function need to be investigated in more detail. It is important to emphasize that the development and reduction of fibrosis is thought to be of paramount importance in recovery of function. Again, the HTX model and its variations remain the most critical preclinical models to study these intricate mechanisms.

## Imaging Modalities and Functional Assessment Technique

While the changes in molecular and cellular remodeling can be assessed well with standard techniques, the correct and reliable assessment of myocardial contractile function is one of the most critical issues remaining to be solved. Because of the characteristic lack of load, the standard methods to visualize cardiac function using echocardiography and catheterization to acquire hemodynamic data mostly fail to provide valid and reproducible data. Here, we report conventional methods and propose innovative ways to assess heart function in the heterotopically transplanted heart.

### Imaging

#### Transabdominal Echocardiography

Because of its non-invasive nature, possibility of repetitive measurements and relative cost-effectiveness, echocardiography is the most common assessment of dimensions and function of the cardiac graft after unloading (72). Small animal echocardiography relies heavily on the use of advanced technology and equipment, such as a 20-MHz transducer for high resolution and imaging quality. The disadvantages of this method include the high variability of acquired data relying on experience of the examiner, well-defined planes of measurement, anatomic variation, and adjustment of technical equipment. Most researchers try to minimize these downsides by repetitive measurements.

#### Magnetic Resonance Tomography

Unlike other cardiovascular imaging techniques, cardiovascular MRI can give access to parameters characterizing morphology, global and regional function, blood flow, myocardial structure, cell damage, metabolism, and other molecular processes in rodents. MRI is rapidly gaining importance as a non-invasive tool for functional and structural assessment of myocardium, with potential new information gained from cine-MRI analysis (9). The necessary know-how and technical equipment for rodent MRI is described in detail in an excellent review (73).

### Hemodynamic and Metabolic Functional Assessment

The HTX model gives us an opportunity to closely check the hemodynamic and metabolic changes in mechanically unloaded myocardium. With advanced imaging techniques, such as positron emission tomography (PET) or MRI, we are able to assess indicators of metabolic changes (7, 9). This will improve our knowledge of dynamic changes induced in the HTX model and help us to understand the complexity and multiple affected structures more adequately.

#### Isolated Cardiomyocyte Function

To distinguish between effects of unloading on the single contractile unit of the heart, the cardiomyocyte in contrast to changes in the organ level mediated by overall shrinking of the heart and myocardial fibrosis and decreased compliance, analysis of the isolated myocyte function is crucial. Shortening of the sarcomeres can be analyzed by videorecording of the contraction and its depiction as a transient, allowing assessment of fractional shortening and relaxation as well as sarcomere length (59, 74).

#### Pressure–Volume Relation

The most rigorous hemodynamic evaluation of cardiac function is acquired *via* conductance catheter insertion generating pressure–volume relationship data (75). Valuable data can be derived from pressure–volume loop analysis in volume-loaded hearts, but the acquisition of pressure–volume loops from volume-unloaded hearts bears intrinsic potential for fault due to the lack of volume itself. This represents an important limitation for this method in heterotopically transplanted, completely unloaded hearts. The tracings from a conductance catheter reveal the almost complete lack of volume in the completely unloaded hearts (9). Another one of the rare studies employing pressure–volume loop analysis in completely and partially unloaded rat hearts to date by Liu et al. (7) reported lower cardiac output, stroke work, and volume in completely unloaded hearts, correlating with lower metabolic activity.

#### Positron Emission Tomography

Atrophic changes in chronically unloaded hearts characteristically include upregulation of the fetal isoform of the glucose transporter GLUT1 (9). Cardiac glucose utilization mirrors work load and thus FDG-PET may be used to assess the metabolic consequences of complete and partial unloading of the heart (9). In two published examinations, FDG-PET was not consistent in revealing differences between unloaded and partially unloaded myocardium (7, 9). There was, however, a correlation between metabolic activity and stroke work, which was lower in completely unloaded hearts compared with partially volume-loaded hearts (7).

#### Isolated Working Heart

After chronic unloading of the left ventricle with either model of hHTX, assessment of the contractile function under reloaded conditions remains a major issue. Still, all translational research efforts aiming at exploitation of the HTX model to mimic the therapeutic effects of unloading rely on such information as the ultimate endpoint. The isolated working heart model represents a possibility to control volume-load and perfusion pressure and thus poses the ideal method to assess the functional capacity of the unloaded heart (76). Although the proper retrieval and subsequent cannulation of the hearts is challenging, this method offers most valuable knowledge of the hearts remaining capacity to sustain under volume reloading.

## Conclusion

This review points to several aspects of unloading as a potentially therapeutic strategy in severe HF: our summary of effects on the organ structure, cellular features, and molecular changes decipher myocardial unloading as an active remodeling process. It shares both aspects of HF and its pathognomonic cellular and molecular features as well as of the reverse remodeling induced by LVADs, with may indicate a recovering heart. So far, the field is still in search of key factors signifying recovery potential. One reason might be the significant differences between the hHTX model and the human setting, which has been addressed by several developments of the model in order to more accurately mimic the unloading of human myocardium, and we have depicted these, offering some advantages. Novel strategies to assess and analyze the unloaded rodent heart will definitely increase the impact of hHTX as a model in the future, spurned by the desire to elucidate the basis of myocardial recovery and advancing it toward a relevant clinical therapeutic strategy.

## Author Contributions

XF and AS reviewed the literature and wrote the manuscript. TC and HT gave idea and conceptual work of the manuscript, edited/reviewed the manuscript. HM gave conception of the topic and manuscript, literature review, and wrote the manuscript.

## Conflict of Interest Statement

The authors declare that the research was conducted in the absence of any commercial or financial relationships that could be construed as a potential conflict of interest.

## References

[1] Kirklin JK Naftel DC Pagani FD Kormos RL Stevenson LW Blume ED Seventh INTERMACS annual report: 15,000 patients and counting. J Heart Lung Transplant (2015) 34:1495–504. 10.1016/j.healun.2015.10.003 26520247

[2] Birks EJ George RS Hedger M Bahrami T Wilton P Bowles CT Reversal of severe heart failure with a continuous-flow left ventricular assist device and pharmacological therapy: a prospective study. Circulation (2011) 123:381–90. 10.1161/CIRCULATIONAHA.109.933960 21242487

[3] Maybaum S Mancini D Xydas S Starling RC Aaronson K Pagani FD Cardiac improvement during mechanical circulatory support: a prospective multicenter study of the LVAD Working Group. Circulation (2007) 115:2497–505. 10.1161/CIRCULATIONAHA.106.633180 17485581

[4] Kinoshita M Takano H Taenaka Y Mori H Takaichi S Noda H Cardiac disuse atrophy during LVAD pumping. ASAIO Trans (1988) 34:208–12. 3196510

[5] Ono K Lindsey ES Improved technique of heart transplantation in rats. J Thorac Cardiovasc Surg (1969) 57:225–9. 4884735

[6] Tevaearai HT Walton GB Eckhart AD Keys JR Koch WJ. Heterotopic transplantation as a model to study functional recovery of unloaded failing hearts. J Thorac Cardiovasc Surg (2002) 124:1149–56. 10.1067/mtc.2002.127315 12447181

[7] Liu Y Maureira P Gauchotte G Falanga A Marie V Olivier A Effect of chronic left ventricular unloading on myocardial remodeling: multimodal assessment of two heterotopic heart transplantation techniques. J Heart Lung Transplant (2015) 34:594–603. 10.1016/j.healun.2014.11.015 25703962

[8] Carr CA Ball D Tyler DJ Bushell A Sykes A Clarke K Varying degrees of ventricular unloading in the heterotopic rat heart transplant model demonstrated by magnetic resonance imaging. Int J Biomed Sci (2014) 10:223–8. 25598751 PMC4289694

[9] Didie M Biermann D Buchert R Hess A Wittkopper K Christalla P Preservation of left ventricular function and morphology in volume-loaded versus volume-unloaded heterotopic heart transplants. Am J Physiol Heart Circ Physiol (2013) 305:H533–41. 10.1152/ajpheart.00218.2013 23771692

[10] Tang-Quan KR Bartos J Deuse T Churchill E Schafer H Reichenspurner H Non-volume-loaded heart provides a more relevant heterotopic transplantation model. Transpl Immunol (2010) 23:65–70. 10.1016/j.trim.2010.04.005 20403439

[11] Klein I Hong C Schreiber SS. Isovolumic loading prevents atrophy of the heterotopically transplanted rat heart. Circ Res (1991) 69:1421–5. 10.1161/01.RES.69.5.1421 1718626

[12] Wang J Tsukashita M Nishina T Marui A Yoshikawa E Muranaka H Chronic partial unloading restores beta-adrenergic responsiveness and reverses receptor downregulation in failing rat hearts. J Thorac Cardiovasc Surg (2009) 137:465–70. 10.1016/j.jtcvs.2008.08.033 19185171

[13] Ibrahim M Navaratnarajah M Kukadia P Rao C Siedlecka U Cartledge JE Heterotopic abdominal heart transplantation in rats for functional studies of ventricular unloading. J Surg Res (2013) 179:e31–9. 10.1016/j.jss.2012.01.053 22520576

[14] Wohlschlaeger J Schmitz KJ Schmid C Schmid KW Keul P Takeda A Reverse remodeling following insertion of left ventricular assist devices (LVAD): a review of the morphological and molecular changes. Cardiovasc Res (2005) 68:376–86. 10.1016/j.cardiores.2005.06.030 16024006

[15] Wang HJ Wang W Cornish KG Rozanski GJ Zucker IH. Cardiac sympathetic afferent denervation attenuates cardiac remodeling and improves cardiovascular dysfunction in rats with heart failure. Hypertension (2014) 64:745–55. 10.1161/HYPERTENSIONAHA.114.03699 24980663 PMC4162756

[16] Fensterer TF Miller CJ Perez-Abadia G Maldonado C. Novel cuff design to facilitate anastomosis of small vessels during cervical heterotopic heart transplantation in rats. Comp Med (2014) 64:293–9. 25296016 PMC4170094

[17] Santamore WP Dell’Italia LJ. Ventricular interdependence: significant left ventricular contributions to right ventricular systolic function. Prog Cardiovasc Dis (1998) 40:289–308. 10.1016/S0033-0620(98)80049-2 9449956

[18] Burkhoff D Klotz S Mancini DM. LVAD-induced reverse remodeling: basic and clinical implications for myocardial recovery. J Card Fail (2006) 12:227–39. 10.1016/j.cardfail.2005.10.012 16624689

[19] Razeghi P Sharma S Ying J Li YP Stepkowski S Reid MB Atrophic remodeling of the heart in vivo simultaneously activates pathways of protein synthesis and degradation. Circulation (2003) 108:2536–41. 10.1161/01.CIR.0000096481.45105.13 14610007

[20] Brinks H Tevaearai H Muhlfeld C Bertschi D Gahl B Carrel T Contractile function is preserved in unloaded hearts despite atrophic remodeling. J Thorac Cardiovasc Surg (2009) 137:742–6. 10.1016/j.jtcvs.2008.09.020 19258100

[21] Depre C Shipley GL Chen W Han Q Doenst T Moore ML Unloaded heart in vivo replicates fetal gene expression of cardiac hypertrophy. Nat Med (1998) 4:1269–75. 10.1038/3253 9809550

[22] Brinks H Giraud MN Segiser A Ferrie C Longnus S Ullrich ND Dynamic patterns of ventricular remodeling and apoptosis in hearts unloaded by heterotopic transplantation. J Heart Lung Transplant (2014) 33:203–10. 10.1016/j.healun.2013.10.006 24315785 PMC3946672

[23] Narula J Haider N Virmani R DiSalvo TG Kolodgie FD Hajjar RJ Apoptosis in myocytes in end-stage heart failure. N Engl J Med (1996) 335:1182–9. 10.1056/NEJM199610173351603 8815940

[24] Haider N Arbustini E Gupta S Liu H Narula N Hajjar R Concurrent upregulation of endogenous proapoptotic and antiapoptotic factors in failing human hearts. Nat Clin Pract Cardiovasc Med (2009) 6:250–61. 10.1038/ncpcardio1452 19234503

[25] Birks EJ. Molecular changes after left ventricular assist device support for heart failure. Circ Res (2013) 113:777–91. 10.1161/CIRCRESAHA.113.301413 23989719

[26] Muranaka H Marui A Tsukashita M Wang J Nakano J Ikeda T Prolonged mechanical unloading preserves myocardial contractility but impairs relaxation in rat heart of dilated cardiomyopathy accompanied by myocardial stiffness and apoptosis. J Thorac Cardiovasc Surg (2010) 140:916–22. 10.1016/j.jtcvs.2010.02.006 20381089

[27] Schena S Kurimoto Y Fukada J Tack I Ruiz P Pang M Effects of ventricular unloading on apoptosis and atrophy of cardiac myocytes. J Surg Res (2004) 120:119–26. 10.1016/j.jss.2003.10.006 15172198

[28] Tsuneyoshi H Oriyanhan W Kanemitsu H Shiina R Nishina T Ikeda T Heterotopic transplantation of the failing rat heart as a model of left ventricular mechanical unloading toward recovery. ASAIO J (2005) 51:116–20. 10.1097/01.MAT.0000150325.05589.8B 15745145

[29] Oriyanhan W Tsuneyoshi H Nishina T Matsuoka S Ikeda T Komeda M. Determination of optimal duration of mechanical unloading for failing hearts to achieve bridge to recovery in a rat heterotopic heart transplantation model. J Heart Lung Transplant (2007) 26:16–23. 10.1016/j.healun.2006.10.016 17234512

[30] Oyabu J Yamaguchi O Hikoso S Takeda T Oka T Murakawa T Autophagy-mediated degradation is necessary for regression of cardiac hypertrophy during ventricular unloading. Biochem Biophys Res Commun (2013) 441:787–92. 10.1016/j.bbrc.2013.10.135 24211573

[31] Hariharan N Maejima Y Nakae J Paik J Depinho RA Sadoshima J. Deacetylation of FoxO by Sirt1 plays an essential role in mediating starvation-induced autophagy in cardiac myocytes. Circ Res (2010) 107:1470–82. 10.1161/CIRCRESAHA.110.227371 20947830 PMC3011986

[32] Zeng Y Cheng L Chen H Cao H Hauser ER Liu Y Effects of FOXO genotypes on longevity: a biodemographic analysis. J Gerontol A Biol Sci Med Sci (2010) 65:1285–99. 10.1093/gerona/glq156 20884733 PMC2990269

[33] Baskin KK Rodriguez MR Kansara S Chen W Carranza S Frazier OH MAFbx/Atrogin-1 is required for atrophic remodeling of the unloaded heart. J Mol Cell Cardiol (2014) 72:168–76. 10.1016/j.yjmcc.2014.03.006 24650875 PMC4037330

[34] Kassiotis C Ballal K Wellnitz K Vela D Gong M Salazar R Markers of autophagy are downregulated in failing human heart after mechanical unloading. Circulation (2009) 120:S191–7. 10.1161/CIRCULATIONAHA.108.842252 19752367 PMC2778323

[35] Wellnitz K Taegtmeyer H. Mechanical unloading of the failing heart exposes the dynamic nature of autophagy. Autophagy (2010) 6:155–6. 10.4161/auto.6.1.10538 19949312

[36] Birks EJ Tansley PD Hardy J George RS Bowles CT Burke M Left ventricular assist device and drug therapy for the reversal of heart failure. N Engl J Med (2006) 355:1873–84. 10.1056/NEJMoa053063 17079761

[37] Mann DL Barger PM Burkhoff D. Myocardial recovery and the failing heart: myth, magic, or molecular target? J Am Coll Cardiol (2012) 60:2465–72. 10.1016/j.jacc.2012.06.062 23158527 PMC3522780

[38] Tsuneyoshi H Oriyanhan W Kanemitsu H Shiina R Nishina T Matsuoka S Does the beta2-agonist Clenbuterol help to maintain myocardial potential to recover during mechanical unloading? Circulation (2005) 112:I51–6. 10.1161/CIRCULATIONAHA.104.525097 16159865

[39] Soppa GK Lee J Stagg MA Felkin LE Barton PJ Siedlecka U Role and possible mechanisms of Clenbuterol in enhancing reverse remodelling during mechanical unloading in murine heart failure. Cardiovasc Res (2008) 77:695–706. 10.1093/cvr/cvm106 18178572 PMC5436743

[40] Navaratnarajah M Siedlecka U Ibrahim M van Doorn C Soppa G Gandhi A Impact of combined Clenbuterol and metoprolol therapy on reverse remodelling during mechanical unloading. PLoS One (2014) 9:e92909. 10.1371/journal.pone.0092909 25268495 PMC4181979

[41] Ambardekar AV Walker JS Walker LA Cleveland JC Jr Lowes BD Buttrick PM. Incomplete recovery of myocyte contractile function despite improvement of myocardial architecture with left ventricular assist device support. Circ Heart Fail (2011) 4:425–32. 10.1161/CIRCHEARTFAILURE.111.961326 21540356 PMC3407673

[42] Navaratnarajah M Ibrahim M Siedlecka U van Doorn C Shah A Gandhi A Influence of ivabradine on reverse remodelling during mechanical unloading. Cardiovasc Res (2013) 97:230–9. 10.1093/cvr/cvs318 23079200

[43] Kurazumi H Li TS Takemoto Y Suzuki R Mikamo A Guo CY Haemodynamic unloading increases the survival and affects the differentiation of cardiac stem cells after implantation into an infarcted heart. Eur J Cardiothorac Surg (2014) 45:976–82. 10.1093/ejcts/ezt629 24459213

[44] Minatoya Y Ito K Kagaya Y Asaumi Y Takeda M Nakayama M Depressed contractile reserve and impaired calcium handling of cardiac myocytes from chronically unloaded hearts are ameliorated with the administration of physiological treatment dose of T3 in rats. Acta Physiol (Oxf) (2007) 189:221–31. 10.1111/j.1748-1716.2006.01636.x 17305702

[45] Pantos C Thyroid hormone at physiological doses restores depressed contractile reserve and impaired calcium handling of cardiac myocytes from chronically unloaded hearts. Acta Physiol (Oxf) (2007) 189:219 10.1111/j.1365-201X.2007.01675_1.x 17305701

[46] Neubauer S The failing heart – an engine out of fuel. N Engl J Med (2007) 356:1140–51. 10.1056/NEJMra063052 17360992

[47] Gupte AA Hamilton DJ Cordero-Reyes AM Youker KA Yin Z Estep JD Mechanical unloading promotes myocardial energy recovery in human heart failure. Circ Cardiovasc Genet (2014) 7:266–76. 10.1161/CIRCGENETICS.113.000404 24825877 PMC4394989

[48] Tian R. Transcriptional regulation of energy substrate metabolism in normal and hypertrophied heart. Curr Hypertens Rep (2003) 5:454–8. 10.1007/s11906-003-0052-7 14594563

[49] Baskin KK Taegtmeyer H. Taking pressure off the heart: the ins and outs of atrophic remodelling. Cardiovasc Res (2011) 90:243–50. 10.1093/cvr/cvr060 21354996 PMC3115281

[50] Cao DJ Jiang N Blagg A Johnstone JL Gondalia R Oh M Mechanical unloading activates FoxO3 to trigger Bnip3-dependent cardiomyocyte atrophy. J Am Heart Assoc (2013) 2:e000016. 10.1161/JAHA.113.000016 23568341 PMC3647287

[51] Razeghi P Young ME Ying J Depre C Uray IP Kolesar J Downregulation of metabolic gene expression in failing human heart before and after mechanical unloading. Cardiology (2002) 97:203–9. 10.1159/000063122 12145475

[52] Young ME Patil S Ying J Depre C Ahuja HS Shipley GL Uncoupling protein 3 transcription is regulated by peroxisome proliferator-activated receptor (alpha) in the adult rodent heart. FASEB J (2001) 15:833–45. 10.1096/fj.00-0351com 11259402

[53] Taegtmeyer H Razeghi P Young ME. Mitochondrial proteins in hypertrophy and atrophy: a transcript analysis in rat heart. Clin Exp Pharmacol Physiol (2002) 29:346–50. 10.1046/j.1440-1681.2002.03656.x 11985548

[54] Bers DM Perez-Reyes E. Ca channels in cardiac myocytes: structure and function in Ca influx and intracellular Ca release. Cardiovasc Res (1999) 42:339–60. 10.1016/S0008-6363(99)00038-3 10533572

[55] Heineke J Molkentin JD. Regulation of cardiac hypertrophy by intracellular signalling pathways. Nat Rev Mol Cell Biol (2006) 7:589–600. 10.1038/nrm1983 16936699

[56] Hill JA Olson EN Cardiac plasticity. N Engl J Med (2008) 358:1370–80. 10.1056/NEJMra072139 18367740

[57] Ito K Nakayama M Hasan F Yan X Schneider MD Lorell BH. Contractile reserve and calcium regulation are depressed in myocytes from chronically unloaded hearts. Circulation (2003) 107:1176–82. 10.1161/01.CIR.0000051463.72137.96 12615798

[58] Schwoerer AP Neef S Broichhausen I Jacubeit J Tiburcy M Wagner M Enhanced Ca(2)+ influx through cardiac L-type Ca(2)+ channels maintains the systolic Ca(2)+ transient in early cardiac atrophy induced by mechanical unloading. Pflugers Arch (2013) 465:1763–73. 10.1007/s00424-013-1316-y 23842739 PMC3898408

[59] Ibrahim M Al Masri A Navaratnarajah M Siedlecka U Soppa GK Moshkov A Prolonged mechanical unloading affects cardiomyocyte excitation-contraction coupling, transverse-tubule structure, and the cell surface. FASEB J (2010) 24:3321–9. 10.1096/fj.10-156638 20430793 PMC2923356

[60] Ibrahim M Navaratnarajah M Siedlecka U Rao C Dias P Moshkov AV Mechanical unloading reverses transverse tubule remodelling and normalizes local Ca(2+)-induced Ca(2+)release in a rodent model of heart failure. Eur J Heart Fail (2012) 14:571–80. 10.1093/eurjhf/hfs038 22467752 PMC3359860

[61] Takaseya T Ishimatsu M Tayama E Nishi A Akasu T Aoyagi S. Mechanical unloading improves intracellular Ca2+ regulation in rats with doxorubicin-induced cardiomyopathy. J Am Coll Cardiol (2004) 44:2239–46. 10.1016/j.jacc.2004.08.057 15582323

[62] Dipla K Mattiello JA Jeevanandam V Houser SR Margulies KB. Myocyte recovery after mechanical circulatory support in humans with end-stage heart failure. Circulation (1998) 97:2316–22. 10.1161/01.CIR.97.23.2316 9639375

[63] Ogletree-Hughes ML Stull LB Sweet WE Smedira NG McCarthy PM Moravec CS. Mechanical unloading restores beta-adrenergic responsiveness and reverses receptor downregulation in the failing human heart. Circulation (2001) 104:881–6. 10.1161/hc3301.094911 11514373

[64] Wang J Marui A Ikeda T Komeda M. Partial left ventricular unloading reverses contractile dysfunction and helps recover gene expressions in failing rat hearts. Interact Cardiovasc Thorac Surg (2008) 7:27–31. 10.1510/icvts.2007.165563 18006556

[65] Travers JG Kamal FA Robbins J Yutzey KE Blaxall BC. Cardiac fibrosis: the fibroblast awakens. Circ Res (2016) 118:1021–40. 10.1161/CIRCRESAHA.115.306565 26987915 PMC4800485

[66] Bruckner BA Stetson SJ Perez-Verdia A Youker KA Radovancevic B Connelly JH Regression of fibrosis and hypertrophy in failing myocardium following mechanical circulatory support. J Heart Lung Transplant (2001) 20:457–64. 10.1016/S1053-2498(00)00321-1 11295584

[67] Rodrigue-Way A Burkhoff D Geesaman BJ Golden S Xu J Pollman MJ Sarcomeric genes involved in reverse remodeling of the heart during left ventricular assist device support. J Heart Lung Transplant (2005) 24:73–80. 10.1016/j.healun.2003.10.016 15653383

[68] Wang WJ Meng ZL Mo YC Liu JW Sun CC Hu SS Unloading the infarcted heart affect MMPs-TIMPs axis in a rat cardiac heterotopic transplantation model. Mol Biol Rep (2012) 39:277–83. 10.1007/s11033-011-0736-z 21559840

[69] Wang L Xu YX Du XJ Sun QG Tian YJ. Dynamic expression profiles of MMPs/TIMPs and collagen deposition in mechanically unloaded rat heart: implications for left ventricular assist device support-induced cardiac alterations. J Physiol Biochem (2013) 69:477–85. 10.1007/s13105-013-0235-x 23315238

[70] Klotz S Foronjy RF Dickstein ML Gu A Garrelds IM Danser AH Mechanical unloading during left ventricular assist device support increases left ventricular collagen cross-linking and myocardial stiffness. Circulation (2005) 112:364–74. 10.1161/CIRCULATIONAHA.104.515106 15998679

[71] Bruggink AH van Oosterhout MF de Jonge N Ivangh B van Kuik J Voorbij RH Reverse remodeling of the myocardial extracellular matrix after prolonged left ventricular assist device support follows a biphasic pattern. J Heart Lung Transplant (2006) 25:1091–8. 10.1016/j.healun.2006.05.011 16962471

[72] Kutschka I Sheikh AY Sista R Hendry SL Chun HJ Hoyt G A novel platform device for rodent echocardiography. ILAR J (2008) 49:E1–7. 10.1093/ilar.49.2.E1 18506056

[73] Makowski MR Wiethoff AJ Jansen CH Botnar RM. Cardiovascular MRI in small animals. Expert Rev Cardiovasc Ther (2010) 8:35–47. 10.1586/erc.09.126 20014933

[74] Soppa GK Lee J Stagg MA Siedlecka U Youssef S Yacoub MH Prolonged mechanical unloading reduces myofilament sensitivity to calcium and sarcoplasmic reticulum calcium uptake leading to contractile dysfunction. J Heart Lung Transplant (2008) 27:882–9. 10.1016/j.healun.2008.05.005 18656802

[75] Pacher P Nagayama T Mukhopadhyay P Batkai S Kass DA. Measurement of cardiac function using pressure-volume conductance catheter technique in mice and rats. Nat Protoc (2008) 3:1422–34. 10.1038/nprot.2008.138 18772869 PMC2597499

[76] Dornbierer M Stadelmann M Sourdon J Gahl B Cook S Carrel TP Early reperfusion hemodynamics predict recovery in rat hearts: a potential approach towards evaluating cardiac grafts from non-heart-beating donors. PLoS One (2012) 7:e43642. 10.1371/journal.pone.0043642 22928009 PMC3424125

